# Integrating multiomics and prior knowledge: a study of the Graphnet penalty impact

**DOI:** 10.1093/bioinformatics/btad454

**Published:** 2023-07-25

**Authors:** Hamza Chegraoui, Vincent Guillemot, Amine Rebei, Arnaud Gloaguen, Jacques Grill, Cathy Philippe, Vincent Frouin

**Affiliations:** Université Paris-Saclay, CEA, Neurospin, 91191 Gif-sur-Yvette, France; Institut Pasteur, Université Paris Cité, Bioinformatics and Biostatistics Hub, 75015 Paris, France; Université Paris-Saclay, CEA, Neurospin, 91191 Gif-sur-Yvette, France; Centre National de Recherche en Génomique Humaine, Institut de Biologie François Jacob, CEA, Université Paris-Saclay, 91000 Evry, France; Département Cancérologie de l’enfant et de l’adolescent, Gustave-Roussy, 94800 Villejuif, France; Prédicteurs Moléculaires et Nouvelles Cibles en Oncologie—U981, Inserm, Université Paris-Saclay, 94800 Villejuif, France; Université Paris-Saclay, CEA, Neurospin, 91191 Gif-sur-Yvette, France; Université Paris-Saclay, CEA, Neurospin, 91191 Gif-sur-Yvette, France

## Abstract

**Motivation:**

In the field of oncology, statistical models are used for the discovery of candidate factors that influence the development of the pathology or its outcome. These statistical models can be designed in a multiblock framework to study the relationship between different multiomic data, and variable selection is often achieved by imposing constraints on the model parameters. *A priori* graph constraints have been used in the literature as a way to improve feature selection in the model, yielding more interpretability. However, it is still unclear how these graphs interact with the models and how they impact the feature selection. Additionally, with the availability of different graphs encoding different information, one can wonder how the choice of the graph meaningfully impacts the results obtained.

**Results:**

We proposed to study the graph penalty impact on a multiblock model. Specifically, we used the SGCCA as the multiblock framework. We studied the effect of the penalty on the model using the TCGA-LGG dataset. Our findings are 3-fold. We showed that the graph penalty increases the number of selected genes from this dataset, while selecting genes already identified in other works as pertinent biomarkers in the pathology. We demonstrated that using different graphs leads to different though consistent results, but that graph density is the main factor influencing the obtained results. Finally, we showed that the graph penalty increases the performance of the survival prediction from the model-derived components and the interpretability of the results.

**Availability and implementation:**

Source code is freely available at https://github.com/neurospin/netSGCCA

## 1 Introduction

The decreasing cost of biological data acquisition technologies has made high-throughput multimodal databases for hundreds of patients publicly available to study clinically relevant problems, especially in oncology. It includes multiomics data such as gene expression profiles, mutation profiles, copy number variants (CNVs), clinical data, imaging data, etc. These different data may be considered as blocks for which each line includes numerous measures for an individual. In a multiblock experimental setting, each individual of all the different blocks are paired across the blocks. This led to the development of new multiblock machine-learning and statistical models designed to integrate data from various sources on the same observations. These statistical models are not only used to predict the outcome of diseases but also to characterize them by finding a set of variables of interest explaining their outcome. However, isolated variables associated with a pathology outcome usually do not by themselves give a biological meaning to this association. For example, at the gene scale, somatic mutation profiles from patients with the same pathology may differ from one to the other because different genes are mutated in the same pathway (Wood *et al.* 2007, Lawrence *et al.* 2014, Le Morvan *et al.* 2017). This leads to the necessity of identifying networks and pathways grouping variables that interact with each other in complex patterns.

Several works have used *a priori* graphs to identify subnetworks of variables of interest (Zhang *et al.* 2017). Some works proposed using graphs as a postanalysis tool to identify the interactions between a set of previously selected variables (Vaske *et al.* 2010, Kim *et al.* 2011, Vandin *et al.* 2011). Others used the graphs as a preprocessing tool, such as smoothing variables over the interaction network (Rapaport *et al.* 2007, Hofree *et al.* 2013, Le Morvan *et al.* 2017). Graphs have also been integrated into statistical models as a penalty over the model parameters. This includes supervised models, such as survival models (Zhang *et al.* 2013) and regression models (Li and Li 2008), and unsupervised models, such as matrix factorization models (Zhu *et al.* 2021).

Our work focuses on studying the effect of the injection of a prior graphical knowledge as a penalty into a parsimonious variant of the Regularized Generalized Canonical Correlation (RGCCA) model (Tenenhaus and Tenenhaus 2011), namely the Sparse Generalized Canonical Correlation Analysis (SGCCA) (Tenenhaus *et al.* 2014). Specifically, we seek to identify the effects of the graph constraint on the variable selection process in a multiblock framework. Comparison with other structured penalties is beyond the scope of this article. First, we review current methods that achieve data integration and prior graphical knowledge addition (Section 2). Second, we present salient aspects of our implementation of the GraphNet penalty in SGCCA (Section 3), named netSGCCA. Third, on a real oncological dataset, we compare the grouping effect and stability of netSGCCA while considering different graphs with different properties and from various bioinformatics sources. And, on the same real dataset, we investigate the relationship between selected features and the disease outcome using survival prediction and pathway enrichment analysis (Section 4).

## 2 Related works

Numerous methods for feature selection have been proposed in the context of multiblock data analysis and it is a challenging issue in multiomics cases where the number of variables exceeds the number of available observations. Feature selection is used to select variables of interest and discard irrelevant variables. This can be done by restricting the search space of the model parameters, specifically by using penalties to impose properties such as sparsity and smoothness. The Least Absolute Shrinkage and Selection Operator (LASSO) (Tibshirani 1996) penalty is generally used to promote sparsity in the parameter estimates. However, this penalty tends to randomly select a few representatives of each group of highly correlated variables, which leads to unstable results. In Zou and Hastie (2005), the authors proposed a solution to this problem by adding a ridge penalty, proposing the so-called Elastic-Net penalty, and showed its grouping property. However, this grouping effect does not necessarily exhibit groups of variables reflecting *a priori* knowledge. For example, it would be desirable to favour the selection of a group of genes interacting with each other to exhibit biological functions and pathways of interest. This would give a more interpretable solution than selecting isolated genes. In this perspective, other variations to the LASSO have been proposed to group the variables, such as the fused LASSO (Tibshirani *et al.* 2005), Octagonal Shrinkage and Clustering Algorithm for Regression (OSCAR) (Bondell and Reich 2008), and group LASSO (Yuan and Lin 2006). However, the fused lasso and OSCAR can be sensitive to the ordering of the variables. Additionally, these methods do not account for the complex patterns of interactions between the variables.

The injection of the *a priori* graph of variable interactions has been proposed to improve the grouping effect and makes it reflect *a priori* knowledge. Li and Li (2008) introduced a penalty in their regression model, using the Laplacian of an *a priori* graph of interactions between variables as an extension of the ridge penalty. They used the normalized graph Laplacian. Their study exhibited a grouping effect property similar to that of Elastic-Net and the capacity of the penalty to find subnetworks in the context of gene expression data. In parallel, Grosenick *et al.* (2013) introduced the same penalty using the un-normalized (‘raw’) graph Laplacian across several sparse regression approaches, naming it GraphNet penalty. They showed the capacity of GraphNet to select more variables than the Elastic-Net in the context of medical imaging. The GraphNet was used in the CCA model with the Absolute value based GraphNet penalty (AGN-SCCA) (Du *et al.* 2016), and the Graph guided pairwise Group Lasso (GGL-CCA) (Du *et al.* 2020) models. These studies also focus on neighbouring but negatively correlated variables and proposed solutions. Finally, GraphNet has been compared with Fused-LASSO (Watanabe *et al.* 2014) and Group-LASSO (Kim *et al.* 2020) and exhibited better performances in terms of selecting meaningful variables.

The GraphNet penalty has been successfully integrated into multiple models aimed at variable selection (Zhang *et al.* 2017) and target prediction (Zhu *et al.* 2021), in the context of genomic data. We specifically chose SGCCA as a multiblock framework because it is suitable for multiomics. This choice is also relevant to study the impact of a prior graph for three reasons. The mathematical formulation of SGCCA naturally allows to add the graph prior as an additional penalty. Recent software implementation of SGCCA using gradient descent strategies presents optimization tools that enable to accommodate the graph prior. Finally, a recent benchmarking paper (Cantini *et al.* 2021) has highlighted the quality of the RGCCA/SGCCA in dimension reduction on which we build.

## 3 Materials and methods

### 3.1 Methods

SGCCA is a combination of RGCCA and $\ell_{1}$ constraint. This constraint promotes sparsity over the selected features to improve the model interpretation. Given *J* blocks $X_{1},\ldots ,X_{J}$ of sizes $n\times p_{1},\ldots ,n\times p_{J}$, respectively, the model aims to optimize the following problem:


(1) $$\begin{array}{c} \underset{w_{1},w_{2},\ldots ,w_{J}}{\text{argmin}}\sum_{\overset{j,k=1}{j\neq k}}^{J}-c_{j,k}\;\text{cov}(X_{j}w_{j},X_{k}w_{k})\\ s.t.\;||w_{j}|{|}_{2}^{2}=1,j=1,\ldots ,J\\ \text{and}\;||w_{j}|{|}_{1}\leq s_{j},j=1,\ldots ,J \end{array}$$

The weights $w_{j}$ are estimated by maximizing the sum of the covariance between pairs of latent components. The $c_{j,k}$ are indicator variables of the link between blocks *j* and *k* and set by the user. The parameters $s_{j}$ define the sparsity level of each block.

We propose to extend the SGCCA to netSGCCA by adding a GraphNet penalty on one block, denoted $X_{g}$, relaxing the $\ell_{2}$ equality constraint and using a gradient descent method to optimize the problem. The extension of our proposal to penalize more than one block is straightforward. Given a graph G=(E,V), we introduce its Laplacian $L_{g}$ into the optimization problem stated in Eq. (1), which then becomes:


(2) $$\begin{array}{c} \underset{w_{1},w_{2},\ldots ,w_{J}}{\text{argmin}}\sum_{\overset{j,k=1}{j\neq k}}^{J}-c_{j,k}\text{cov}\left(X_{j}w_{j},X_{k}w_{k}\right)+\frac{\gamma_{G}}{\lambda_{\text{max}}}w_{g}^{⊤}L_{g}w_{g}\\ s.t.\;{\left\|w_{j}\right\|}_{2}^{2}\leq 1,j=1,\ldots ,J\\ \text{and}\;{\left\|w_{j}\right\|}_{1}\leq s_{j},j=1,\ldots ,J \end{array}$$

Since we want to work with different versions of the graph G, we scaled the graph penalty hyperparameter using $\lambda_{\text{max}}$, the largest eigenvalue of the graph Laplacian, which is a semidefinite symmetric matrix. This was done, because the largest eigenvalue of the graph Laplacian $L_{g}$, whose value is directly related to the density of the graph, is an upper bound for the graph penalty (and respectively, for the gradient of the graph penalty). Indeed, $w_{g}^{⊤}L_{g}w_{g}\leq \lambda_{\text{max}}{\left\|w_{g}\right\|}_{2}^{2}\leq \lambda_{\text{max}}$, when the constraints are satisfied. Scaling the penalty makes the user chosen hyperparameter $\gamma_{G}$ comparable between different graphs and eases the results presentation.

As established by Witten *et al.* (2009), the $\ell_{2}$ inequality constraints is equivalent to an equality constraint if the $s_{j}$ values lead to a solution such that ${\left\|w_{j}\right\|}_{2}^{2}\geq 1$. We chose to maintain the inequality constraint formalism in Equation (2) to keep the convexity of the problem definition. In addition, if a solution satisfies both the $\ell_{1}$ and $\ell_{2}$ constraints, $s_{j}$ must be between 1 and $\sqrt{p_{j}}$.

The problem expressed in Equation (2) belongs to a class of multiconvex optimization problems with nonsmooth constraints. The feasibility of the using of the GraphNet penalty with the SGCCA framework has already been presented by Guigui *et al.* (2019). Multiple solvers for this problem have been proposed and studied (Hadj-Selem *et al.* 2018). We used Löfstedt *et al.*’s (2016) algorithm that updates the parameters $w_{k}$ for each block in turn, while keeping the others fixed, in a cyclic manner. For each block, FISTA (Beck and Teboulle 2009) was used as a gradient descent optimization algorithm. FISTA processes the constraints using their defined projectors. Multiple methods have been proposed to project on a set of convex constraits such as the ‘alternating projections’ or the Dysktra projections. We chose to work with the latter as it converges to the nearest point satisfying both conditions (Bauschke and Borwein 1994). To project onto the $\ell_{1}$ sphere, we used the soft-thresholding operator, while to project onto the $\ell_{2}$ sphere, we used a simple $\ell_{2}$ normalization.

Simulated data were generated to evaluate the netSGCCA method, but for reasons of brevity, their description and related conclusions are given in the Supplementary Materials. A summary of the findings of this simulation is given at the beginning of Section 4.

### 3.2 Real oncological dataset

For this article, we worked with the Low-Grade Glioma (LGG) dataset from The Cancer Genome Atlas (TCGA) project (http://cancergenome.nih.gov). We used the data as they have been made available on https://www.openml.org/ by Herrmann *et al.* (2020). It comprises five groups of variables (five blocks), including CNVs, microRNA expression (miRNA), gene expression (mRNA), mutations, and clinical records, obtained for 419 patients. We did not consider the mutations block for this work because of their binary nature. Multiple works have been proposed to process the binary mutations (Hofree *et al.* 2013, Le Morvan *et al.* 2017) but this goes beyond the scope of this work. Clinical data have also been removed since we are mostly interested in the variable selection from the omic subset.

## 4 Experiments and results

A two-block simulated dataset was built to assess netSGCCA (see Supplementary Material). For one block, the variables were sampled from a distribution defined with a given graph derived covariance matrix. netSGCCA is evaluated by using the same graph as the GraphNet penalty for this block. We observed that netSGCCA outperformed SGCCA in retrieving the variables of interest (see Supplementary Material  Tables S1 and S2). Additionally, it appeared that the graph structure influences the model behaviour. In this section, we further investigate these results on real oncological data.

To assess the behaviour of the proposed method on real data, we used the TCGA-LGG dataset of 419 patients. All patients have the CNV block, which has 57 964 variables, the miRNA with 645 variables, and the mRNA with 22 297 variables. We look for learned features that not only maximize the correlation between different blocks but are also relevant to survival. To do so, we added the null deviance residuals, as a fourth block, computed using the survival data (and not the survival data itself, for the sake of efficiency) to these three blocks, as proposed by Bastien (2008). The addition of the null deviance residuals is only done during the training phase. We applied the GraphNet penalty on the mRNA block. Gene identifiers were mapped to their HUman Genome Organisation (HUGO) names with nonmatching genes removed, leading to 19 864 genes remaining in the mRNA block.

Different gene–gene interaction graphs with different properties were used. Pathway Commons v12 (PC) (Cerami *et al.* 2011) is an aggregation of multiple subgraphs from various sources, containing 15 710 nodes and 841 690 edges. The Molecular Signature Data Base (MSIGDB) (Subramanian *et al.* 2005, Liberzon *et al.* 2011) C3 regulatory target gene set is one of the subgraphs of the PC graph containing 10 463 nodes and 82 962 edges. The Kyoto Encyclopedia of Genes and Genome (KEGG) (Kanehisa and Goto 2000) is also another subgraph of the PC graph, containing 776 nodes and 11 963 edges. The full description of these graphs is found in Table 1. Since graphs do not include all genes, missing genes were added as isolated nodes, and genes mapped to the same HUGO name were duplicated in the graph with their edges.

**Table 1.** Different sources of prior knowledge graphs.

	# nodes	# edges	Diameter	Radius	% isolated nodes
PC	15 710	841 690	6	4	0.20
MSIGDB	10 463	82 962	6	4	0.46
KEGG	776	11 963	12	7	0.96

**Figure 1.**
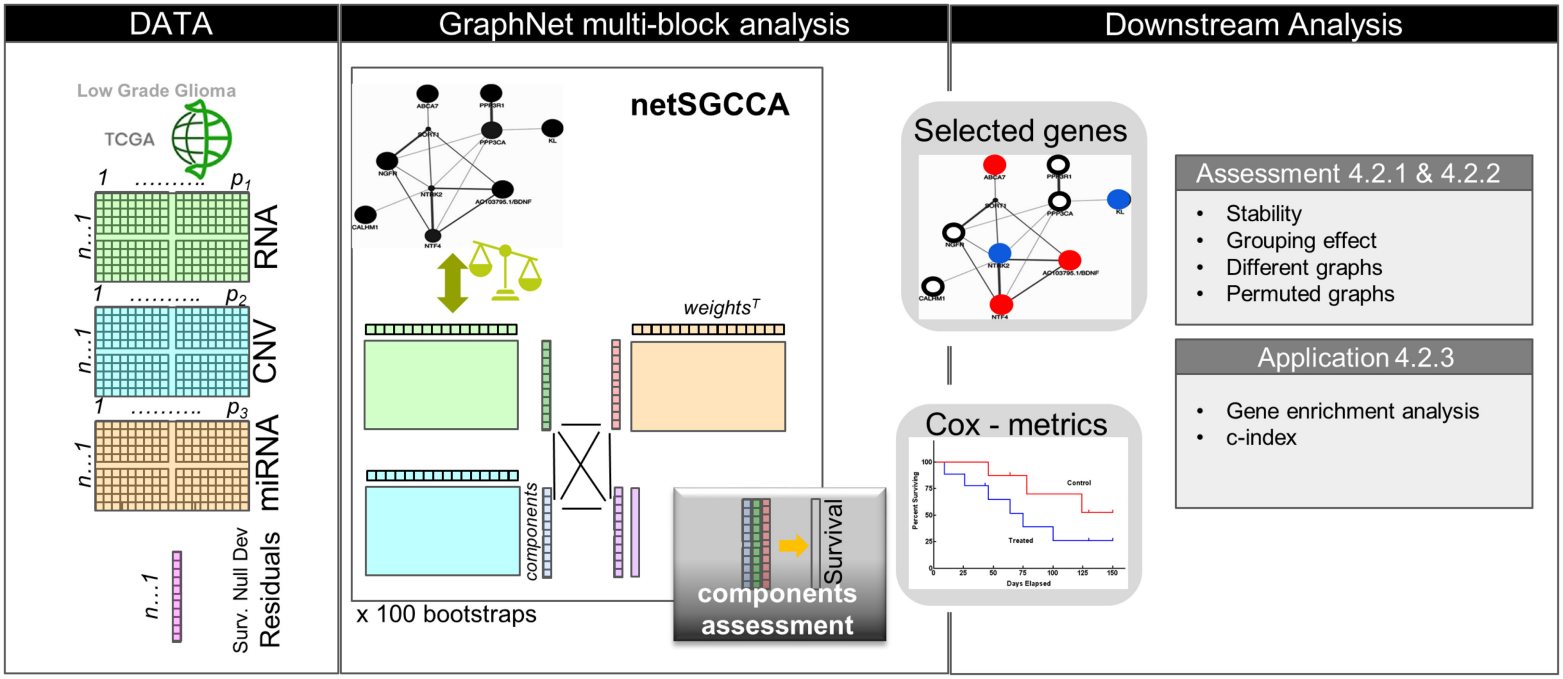
Details of the proposed experimental design for the TCGA-LGG dataset. The analysis step refers to the comparison between the different graphs, analysis of the variable selection when nodes are permuted or edges are removed. Application refers to survival prediction and enrichment analysis.

We aimed to compare the gene selection abilities of the different graphs. First, using the PC graph, we established the differences between the raw and normalized graph Laplacian, in terms of the number of selected variables, as the $\gamma_{G}$ varies, and the stability of variable selection. Then, we compared the different graphs. Later, to establish the impact of the graph in the selection process and disentangle the graph penalty effect from the sparse penalty effect, we permuted the nodes of the PC graph and compared the results with the nonmodified graph. We also removed edges from the PC graph to investigate the effect the graph density and the importance of the edges between selected nodes. Finally, we evaluated the application of the model-derived extracted components by using them to predict survival and discussed the biological pathways potentially involved through the selected genes. Details of the experimental design are presented in Fig. 1 and Supplementary Material  Fig. S2.

In order to achieve the experimental design, we first isolated 15% of the patients (63 patients) as a test set and performed the analysis on the remaining 356 patients forming the training set. We stratified the patients using the event status. We used 100 bootstrap samples without replacement, each containing 85% of the training set. Based on these samples, we performed 100 runs that allowed us to assess the stability of the method and provide the mean to study the distribution of the different metrics used. The test set was only used for the survival prediction in the final part of our study.

Throughout this work, $c_{j,k}=1$ for all *j* and *k*, with j≠k. This means that each block is connected to all the other blocks in the netSGCCA model. Additionally, the $\ell_{1}$ constraints have been fixed as the best yielding parameters – in terms of the *c*-index – on a 5-fold (using the training set) grid search using SGCCA.

### 4.1 Comparison between normalized and raw graph Laplacian

**Figure 2.**
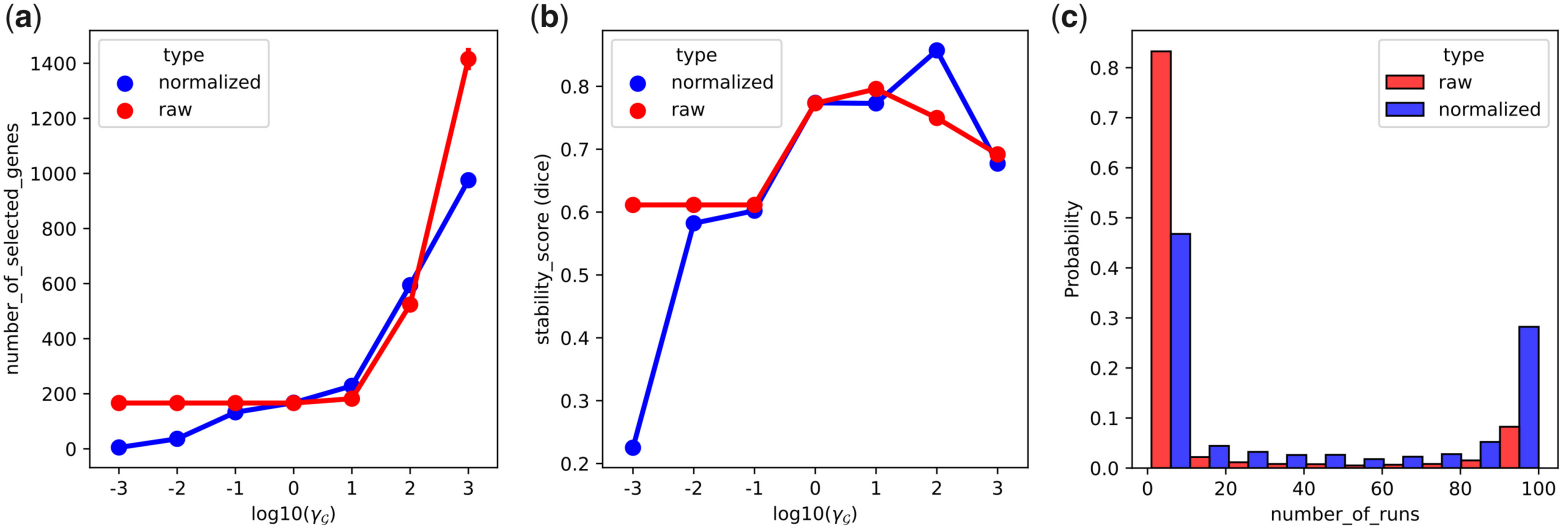
Comparison between raw and normalized graph Laplacian. (a) Evolution of the number of selected genes as $\gamma_{G}$ varies, using the raw and normalized graph Laplacian. (b) Stability assessment using the Dice metric as $\gamma_{G}$ varies, using the raw and normalized graph Laplacian. (c) Distribution of the selection rate of the genes selected at least once in the 100 runs, with $\gamma_{G}=10^{3}$.

To compare the normalized graph Laplacian with its raw version, using the PC graph, we varied the $\gamma_{G}$ between $10^{-3}$ and $10^{3}$. Figure 2a shows the evolution of the number of selected genes, for both graph Laplacians, as $\gamma_{G}$ increases. We can see that the higher the $\gamma_{G}$, the more genes are selected; this is in line with previous findings in the related works. Without a graph penalty, very few genes are selected (about three in each sample). Figure 2a also highlights a similar behaviour between the raw and the normalized graph Laplacian, in terms of the number of genes selected. Furthermore, we assessed the stability of the method, using all pairs of the 100 runs outcomes, by computing the average pairwise Dice similarity coefficient (Zucknick *et al.* 2008) and the normalized percentage of overlapping genes (nPOG) (Zhang *et al.* 2009) as proposed by Nogueira *et al.* (2018) (Supplementary Material  Fig. S2A). Results are shown in Fig. 2b and Supplementary Material  Fig. S3. The Dice metric indicates that models using either graph Laplacians have similar stability profiles. Meanwhile, the nPOG metric indicates that using the normalized graph Laplacian is more stable as $\gamma_{G}$ increases. However, a closer look shows that, using the raw graph Laplacian, 25% of genes were selected only once across the 100 runs, while this is only true for 3% for the normalized as shown in Fig. 2c.

**Figure 3.**
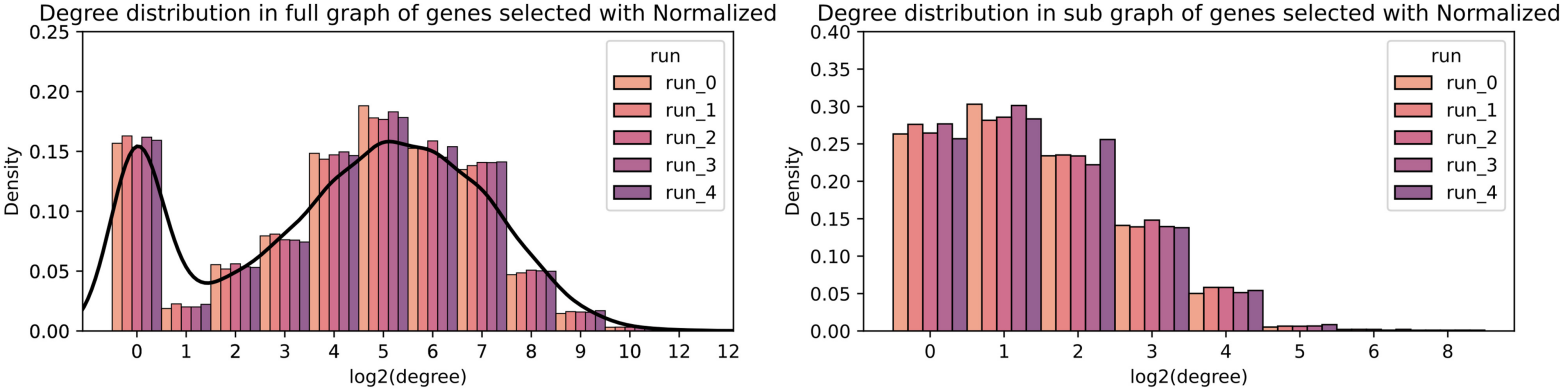
Degree distribution of selected genes in five random runs for normalized graph Laplacian. On the left, for each selected gene, we counted the number of its neighbours in the PC graph. The black line represents the density of the degree distribution of all genes in the PC graph. On the right, for each selected gene, we counted the number of its neighbours among selected genes.

In each of the 100 runs, we examined the degree distributions by considering the subset of selected genes in the full PC graph on the one hand and, on the other hand, in the PC subgraph containing the selected genes. Figure 3 shows the degree distribution of selected genes from five random runs in the full PC graph (left plot), and in the PC subgraph of the selected gene (right plot), using the normalized graph Laplacian. The degree distribution of the selected genes is similar to that of all genes in the PC graph. Moreover, the inspection of the proportion of the isolated nodes shows that they are underrepresented among the selected genes. This shows that GraphNet does not favour genes with a high degree in the graph but discriminates against isolated nodes. Additionally, GraphNet selected few neighbours in the graph. The right plot of Fig. 3 shows a shift towards lesser degrees indicating that genes are mostly selected because of their covariance similarity across the patients, but not because of their neighbourhood in the graph penalty (shown later). The GraphNet penalty, in our configuration, mainly smooths weights over similarly correlated genes but does not give rise to gene communities because of their adjacency in penalty graph. Both the normalized and raw graph Laplacian follow the same behaviour, as exhibited in Supplementary Material  Fig. S4.

**Figure 4.**
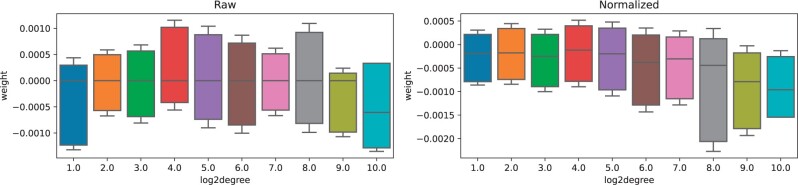
Box plot of the weight distribution of selected genes for raw and normalized graph Laplacians. *x*-Axis is log2 of the gene degrees in full PC graph.

**Figure 5.**
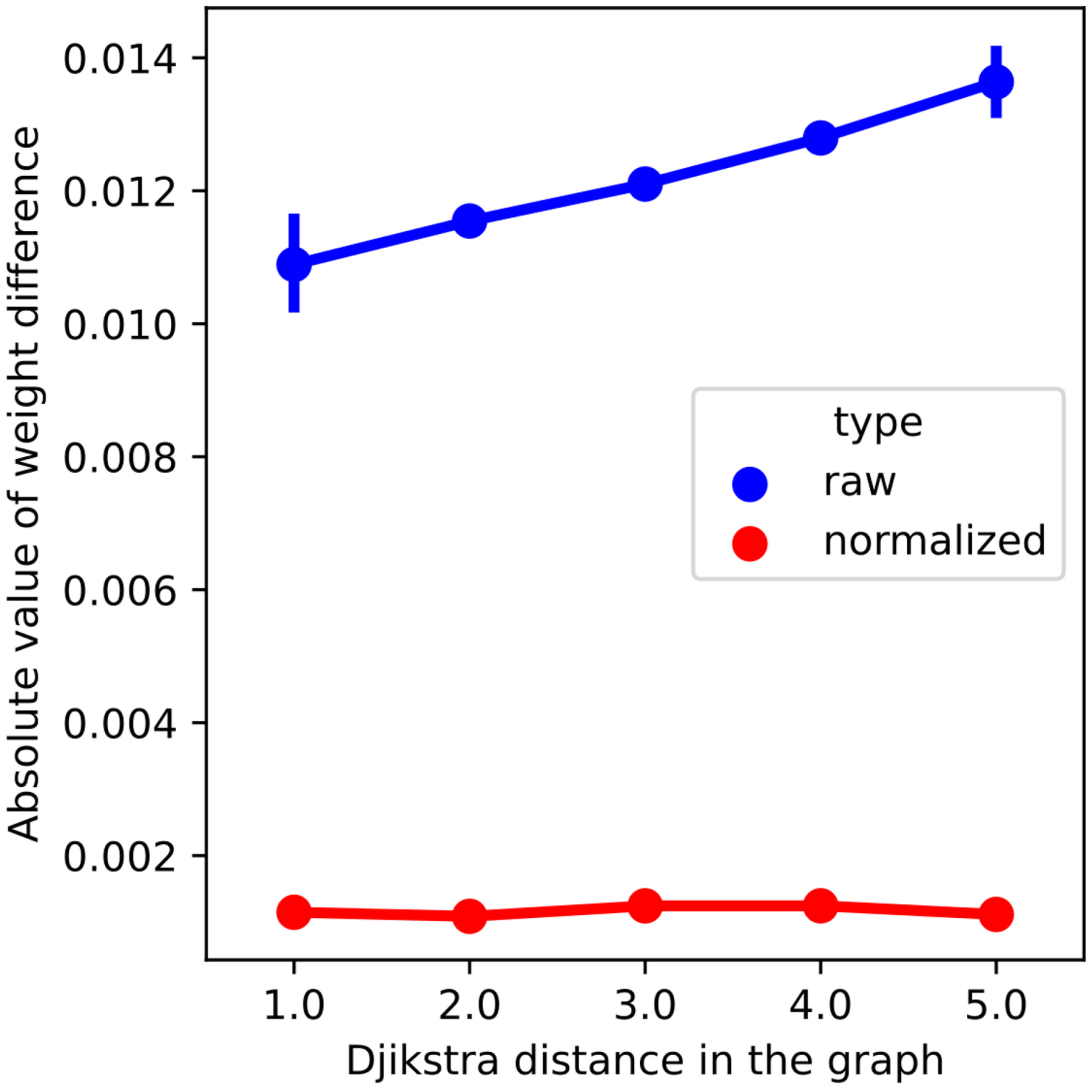
Average absolute weight difference between selected nodes according the Dijkstra distance between them in the PC graph.

We looked at netSGCCA gene weight distribution according to the degree of the gene in the PC graph. Figure 4 shows that the gene degree does not influence the weight. However, isolated nodes tend to have significantly higher weight variance (box not shown in figure), ranging from −0.06 to 0.06 for the normalized graph Laplacian. This is expected as the more a node is connected, the more its weight is constrained. The same pattern can be shown for the normalized and raw graph Laplacian, except that the normalized graph Laplacian produced smaller weights in absolute value. Additionally, Fig. 5 shows the weight difference between genes according to the distance between the genes in the graph (we used the shortest path in the graph). It shows that the closer the genes, the closer their final weights, going from 0.0011 on average between neighbouring nodes to 0.0013 if the shortest path between them is five, for the normalized graph Laplacian. The same pattern can be seen on the raw graph Laplacian with higher values. Thus, even if the graph does not select subnetworks, it has a grouping effect.

Finally, the model selects genes by correlation, which is exhibited in Supplementary Material  Fig. S5, which shows correlation distribution among selected genes by the normalized graph Laplacian. The model selected two distinct, negatively correlated groups. The correlation distribution between selected genes does not follow the distribution of the correlation among gene expression profiles in the whole LGG dataset. The GraphNet penalty partially overrides the $\ell_{1}$ penalty and allow the model to select groups of highly correlated variables.

### 4.2 Comparisons between different graphs

Since the normalized graph Laplacian allowed the selection of more stable gene sets, the following experiments will use only normalized graph Laplacians. We ran our model using the MSIGDB and KEGG graphs on the same 100 bootstrap samples and the same model hyperparameters. We compared the set of selected genes from each sample when using netSGCCA with the normalized PC-graph Laplacian and the normalized Laplacian of the other graphs (see Supplementary Material  Fig. S2B). To do so, we computed the Dice and nPOG metrics between the selected variables. Supplementary Material  Table S3 summarizes the obtained results. It shows that both graphs selected fewer genes than the PC graph, with the MSIGDB selecting more than the KEGG. However, there is a large overlap between the selected genes given each graph penalty. This demonstrates that the graph density strongly influences the grouping effect of the method, but that the choice of the graph does not substantially impact the set of genes selected, as long as the graphs are biologically relevant. Supplementary Material  Fig. S6 shows a Venn diagram of the most frequently (in over 80 samples) selected genes. We note that the genes selected by the KEGG graph are included in the genes selected by the MSIGDB, even though these graphs are independent.

To better exhibit the influence of the graph semantic (information embedded in the graph), we randomly permuted gene labels within the PC graph. This was done ten times to check the validity of the results. We compared, through the Dice metric, the different selected gene sets using the original PC graph and its permuted version for each bootstrap sample. The different permuted graphs selected a similar number of genes as the original PC graph, but as shown in Supplementary Material  Table S4 and Fig. S7, the Dice indices were <0.1. Note that, as shown in Supplementary Material  Tables S3 and S4, the MSIGDB and KEGG graphs selected fewer genes but with higher Dice scores. This shows that the graph semantics strongly influences the results obtained.

**Figure 6.**
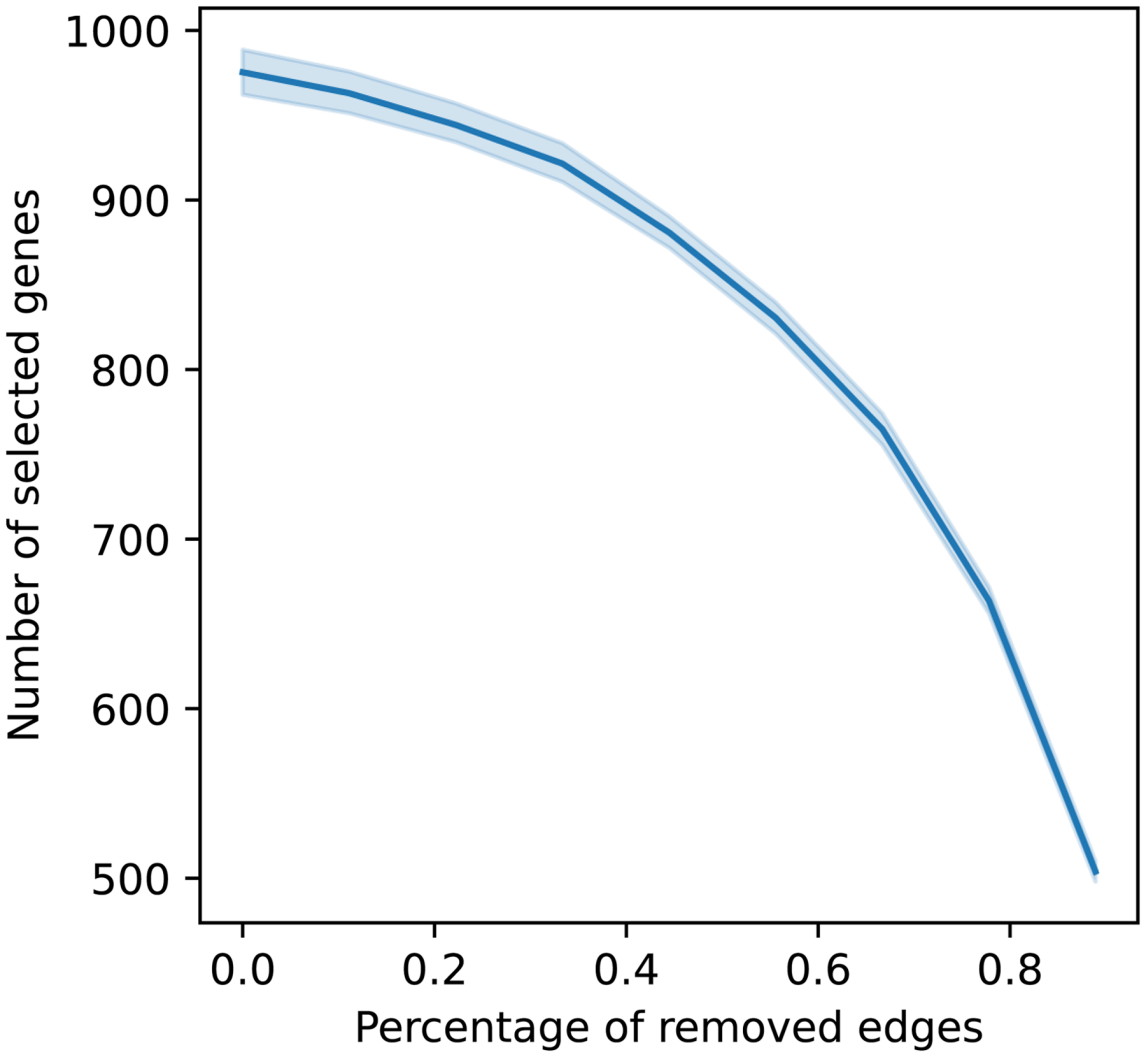
The evolution of the number of selected genes when the number of edges decreases.

To show the impact of the graph density on the number of selected genes, we removed edges randomly from the PC graph. We made sure that, for each bootstrap sample, we removed edges from selected genes using the normalized PC graph in the same proportions as from the whole edge set. Figure 6 shows the evolution of the number of selected genes as edges are removed. It exhibits a strong correlation between graph density $\left(\frac{\#\text{of}\;\text{edges}}{\#\text{of}\;\text{all}\;\text{possible}\;\text{edges}}\right)$ and the number of selected genes. Additionally, selected genes after the removal of edges are all included in the original set of selected genes.

We investigated the importance of the direct and the indirect paths between the genes selected by a model with the GraphNet penalty based on the full PC graph. To do this, for each sample, we removed inner edges (edges connecting two selected genes by the PC graph), outer edges (edges connecting a selected and nonselected gene), and all edges of the selected nodes, which would make them completely isolated. As shown in Supplementary Material  Table S5, removing the inner edges (2431 edges on average) did not change either the number or the set of genes selected. Removing the outer edges (82 966 edges on average) diminished the number of selected genes while keeping a large overlap. Making all the originally selected genes isolated nodes reduce the number of selected genes going from 975 to 169 on average. Even when removing either the inner or the outer edges, some paths may remain between the selected genes, which help the model to retrieve them. However, making them isolated lowers their probability of being selected.

### 4.3 Survival prediction

Using the components extracted from the CNV, mRNA, and the miRNA, by the netSGCCA method, we performed a survival prediction using a simple Cox model. We compared the results using the different graphs and a model without a graph constraint, and we present the results in Table 2. It shows that models using the GraphNet penalty have similar *c*-index scores compared with the model without a graph. When using the MSIGDB and KEGG, results are slightly better compared with the other models on the validation and test sets. This indicates that the GraphNet penalty did not weaken the ability of the estimated components to predict patients’ survival. However, the model without GraphNet could not reliably extract genes, which hindered its ability to identify pathways of interest. SGCCA, without a graph, selected about three variables per fold. It is in line with the results found so far, as the $\ell_{1}$ penalty tends to select few representatives among highly correlated variables. GraphNet forced the model to gather these correlated variables. Using the different graphs for the GraphNet penalty did not change the *c*-index noticeably, which is also expected as the variable selection has shown a substantial overlap. Adding the graph made the signatures from other blocks less stable. The more stable mRNA variables are selected, and the fewer and less stable variables are observed from the CNV block. As a result, no stable variables are obtained from the CNV block when a graph was used, but it must be balanced by the fact that for each fold, the model selected about 150 variables when the normalized graph Laplacian was used (compared to 250 when no graph was used).

**Table 2.** Performances in survival prediction, number of selected variables, and pathways depending on the type of graph to constrain the model.

	*c*-Index		Frequently selected variables in block			No. of pathways from the mRNA
	Validation set	Test set	mRNA	MiRNA	CNV	
No graph	0.708±0.122	0.69±0.04	0	1	109	0
Raw PC	0.692±0.127	0.64±0.06	973	1	0	3
Normalized PC	0.709±0.141	0.72±0.07	499	0	0	3
Normalized MSIGDB	0.741±0.092	0.7±0.07	293	0	0	2
Normalized KEGG	0.712±0.110	0.69±0.05	159	1	97	2

We used the *Enrichr* platform (Chen *et al.* 2013, Kuleshov *et al.* 2016, Xie *et al.* 2021), with the C6 collection to investigate associations between signatures and the 423 selected genes. The results are presented in Table 3. We found pathways that have already been associated with low-grade gliomas. Genes associated with astrocytes in the set CAHOY ASTROGLIAL, have also been selected by the model. These genes has previously been studied for their link with brain tumour development (Katz *et al.* 2012, Irvin *et al.* 2017). *ATF2* is known to promote invasion in malignant glioma (Zhang *et al.* 2015). Additionally, *TGF-*β (Transforming growth factor-beta) has been targeted to limit brain tumour growth (Han *et al.* 2015). Other pathways have been labelled byl enrichment analysis but the corrected *P*-value was not significant. For example, the *RAF* Fusion has been associated with paediatric low-grade tumours (Lind *et al.* 2021). Mutations in *KRAS*, *HRAS* and *NRAS* are known in gliomas and are often concomitant with *BRAF* mutations and fusions (Knobbe *et al.* 2004).

**Table 3.** Top gene sets from GSEA C6 collection.^a^

Term	Adjusted *P*-value
**RAF UP.V1 DN**	.011950
**CAHOY ASTROGLIAL**	.020674
**ATF2 UP.V1 DN**	.049366
TGFB UP.V1 DN	.13655
KRAS.KIDNEY UP.V1 UP	.13655

a In bold, gene sets with an adjusted *P*-value <.05.

## 5 Discussion

Our work establishes some characteristics of netSGCCA, a data integration method that implements the GraphNet penalty. It shows, in the context of multiblock analysis, that GraphNet helps to group variables instead of selecting a few candidates, which is in line with previous results involving the same penalty (Li and Li 2008, Grosenick *et al.* 2013, Watanabe *et al.* 2014). We also exhibit better interpretation capabilities for netSGCCA compared with SGCCA, as it allows the selection of sound and stable candidate variables within a block.

From the application of netSGCCA to a real multimodal oncological dataset, we have derived general observations. For the block being submitted to the GraphNet penalty, the similarity of the variable profiles is the primary driver for coselecting variables, and the proximity of the variables (as nodes) in the graph is secondary. The density of the graph *a priori* used in the GraphNet strongly influences the final number of selected variables. The denser the graph, the more variables were selected. Yet, our results also show that the capacity of the netSGCCA at extracting variables of interest capitalizes mainly on the variables initially selected by the SGCCA (without a graph). Finally, the overlap between selected variables in the penalized block when using graphs with equivalent density but with different semantics, demonstrates the netSGCCA model relies on the graph.

Regarding the LGG pathology, as it may be studied from the TCGA multimodal dataset, we demonstrate two remarkable achievements of netSGCCA that overrides the current performance of other multimodal integration frameworks. First, netSGCCA predicts survival very well. Large *c*-index values were obtained without using the medical data (no eCRF information other than survival). The results found are similar to reference values reported in a recent work which did consider medical data (Herrmann *et al.* 2020). Second, using netSGCCA with the PathwayCommon graph penalty for the gene expression block, we took advantage of the stability of variable selection to propose a list of candidate genes and biological pathways that explain the pathological outcome. Overall, this shows that graph penalty in multimodal analysis model like netSGCCA is able to bring original molecular biology insights into the pathology.

Our results have been largely confirmed on other tumours, including TCGA-KIRP, TCGA-PAAD and TCGA-OV (Supplementary Material Section S3). However, this study is limited to applications in oncology with data from the TCGA. Finally, while a general observation is that GraphNet selects more variables than the classical Elastic-Net, discussions remain about its stability when there are multiple variables of interest with no correlation between them. It is a data-related problem, and the model performance on the studied datasets is insufficient to give indications about its behaviour in such a case.

## 6 Conclusion

The present work focuses on the analysis of the GraphNet penalty available in the multiblock netSGCCA model and applied to the TCGA-LGG dataset. Contrary to Elastic-Net alone, GraphNet penalty is able to select a reasonable set of genes and yields informative biological interpretation from the pathway enrichment analysis. The example on the TCGA-LGG dataset exhibits the stability and reliability of netSGCCA for selecting variables of interest. However, it is important to note that we show that the coselection of variables is not primarily influenced by the structure of the graph, but rather by its overall density. Therefore, an interpretation in terms of the paths read in the graph is illusive. Nevertheless, the method did extract genes that have been found co-(de)regulated in other studies of low-grade gliomas and other brain tumours. Future applications should focus on extending the results to other tumour types. Additionally, the multiblock model should enlarge the scope of multimodality beyond molecular data. New data sources can be investigated, such as imaging data with their specific penalty resources, in order to increase prediction performance or unveil shared information between modalities.

## Supplementary Material

btad454_Supplementary_Data

## Data Availability

For this paper, we worked with the Low-Grade Glioma (LGG) dataset from The Cancer Genome Atlas (TCGA) project (http://cancergenome.nih.gov). We used the data as they have been made available on https://www.openml.org/ by Herrmann et al. (2020).
