# Supplementary material for: Integrating multiomics and prior knowledge: a study of the Graphnet penalty impact
Source: Bioinformatics. 2023 Jul 25;39(8):btad454. doi: 10.1093/bioinformatics/btad454 (PMC10403429; doi:10.1093/bioinformatics/btad454)
Supplement: btad454_Supplementary_Data [file btad454_supplementary_data.pdf]

Integrating multi-omics and prior knowledge : a study of the Graphnet penalty impact

Contents

1 Simulation Study **1**

2 TCGA-LGG: Additional Results **6**

3 Additional datasets **12**

3.1 Comparison between normalised and raw graph Laplacian . . 12

3.2 Comparisons between different graphs 13

1 Simulation Study

We tested netSGCCA using two simulated blocks $\mathbf{X}_1$ and $\mathbf{X}_2$ with a graph penalty based on a graph $\mathcal{G}$ on the second block.Our simulation procedure followed Du et al. [1] proposal. We started by defining the vectors $\mathbf{u}_1$ and $\mathbf{u}_2$ of dimensions $p_1 = 150$ and $p_2 = 101$. Then we generated $n = 80$ samples for each row of the two blocks $x_1|z \sim \mathcal{N}\left(cz\mathbf{u}_1^\top, \Sigma_1\right)$ (respectively $\mathbf{x}_2|z \sim \mathcal{N}\left(cz\mathbf{u}_2^\top, \Sigma_2\right)$), with $z \sim \mathcal{N}(0,1)$ a latent variable. We defined $(\Sigma_1)_{kl} = 0.1$, and $(\Sigma_2)_{kl} = -0.9 \times |u_k - u_l| + 0.9$ if the variables k and l are adjacent in the graph $\mathcal{G}$, and 0.1 otherwise. The variance of each vector is 1. The vector $\mathbf{u}_1 = (\underbrace{0, \cdots, 0}_{60}, \underbrace{1, \cdots, 1}_{30}, \underbrace{0, \cdots, 0}_{60})$ was used in all configurations. Finally, the coefficient $c \in \{0.5, 2\}$ was used to simulate data with different mean/variance ratios. Since the defined correlation matrices are not necessarily positive semi-definite, we used the nearest correlation matrix as proposed by [2].

We tested 12 different configurations, each configuration consists of a vector $\mathbf{u}_2$ and a graph. The different configurations allow us to assess the properties of the method on diverse situations. As a remainder, the aim of the simulation is not to compare the different graphs, as they are expected to be *a priori* knowledge and not computed. But, we aim to assess the behaviour of the different graph types in the different cases.

Three different cases of $\mathbf{u}_2$ were used to simulate different interaction types between variables of interest. The first case, $\mathbf{u}_2 = (\underbrace{0, \cdots, 0}_{40}, \underbrace{1, \cdots, 1}_{20}, \underbrace{0, \cdots, 0}_{41})$. The second case, $\mathbf{u}_2 = (\underbrace{0, \cdots, 0}_{40}, \underbrace{1, \cdots, 1}_{10}, 0, \underbrace{-1, \cdots, -1}_{10}, \underbrace{0, \cdots, 0}_{41})$. And the third case, $\mathbf{u}_2 = (\underbrace{0, \cdots, 0}_{41}, \underbrace{1, -1, 1 \cdots, -1, 1}_{20}, \underbrace{0, \cdots, 0}_{40})$. Additionally, four different graphs were investigated, the path (where the edges are between subsequent variables), the star graph (where the 50th variable is connected to all the others), the union of the path and the star graph and finally the complete graph. An illustration of the different cases car be found at Figure S1.

The model performance was assessed using the correlation between the estimated components. Additionally, we computed the precision, recall and F1 metrics between the true $\mathbf{u}_j$ vectors and the weights $\mathbf{w}_j$ estimated by the model. For each configuration, we chose the hyper-parameter $\gamma_{\mathcal{G}}$ by running the model 20 times, with $\gamma_{\mathcal{G}}$ ranging from 10^{-4} to 10^4 each time. The best $\gamma_{\mathcal{G}}$ was selected using the best average F1 score because our objective is mainly to recover the variables of interest. For each configuration, we also ran the model without using a graph and compared the results. The sparsity value was fixed to $\sqrt{25}$ for the first block (resp. $\sqrt{20}$ for the second block) in all runs.

Table S1 and Table S2 show the results obtained on the simulated data. It shows that when we have a high mean/variance ratio, the F1 scores are higher, which means that the models using the graphs focus on retrieving the underlying projector $\mathbf{u}_2$. This was expected since a low mean/variance ratio means noisier data.

Overall, the tables also show that using netSGCCA outperformed the SGCCA without a graph. When $c = 2$, SGCCA selected very few variables, about 3, leading to a high precision but very low recall. In contrast, the graph penalisation allowed the model to select more variables, retrieving all the variables of interest and a high F1 score. However, this increase of the F1 score came with a slight decrease in the correlation between the estimated

components, by around 2%. Additionally, when $c = 0.5$, the F1 score continues to show improvement when the graph penalisation is used, but to a lower degree. However, the correlation between the estimated components also increased by 0.13 on average.

Comparing each graph type, we can see that, when $c = 2$, the path graph recovers all the variables of interest perfectly, with an average F1 score of 1 in all cases. The star graph was also able to obtain a perfect recall but with much lower precision. This is because the star graph selects many more variables, about 45 on average. Knowing that the sparsity level is the same for all configurations, the hub in the star graph seems to spread the weights more into its neighbours compared to the path graph. However, the correlations between estimated components are comparable. When $c = 0.5$, the models seem to select the variables randomly, which is shown by an F1 score close to 0.2. Additionally, the weights do not seem to resemble the original $\mathbf{u}_2$. However, even this result is better than without the graph *a priori*, which only selected a couple of features and resulted in an F1 score close to 0. In this situation, by choosing a greater number of variables, the star graph performed better in the correlation score compared to the path graph. Additionally, the union of the star and path graph exhibited behaviour similar to the ones of path and star graphs. Finally, the models with the complete graph always failed to outperform all the other models. This result is expected since the complete graph does not contribute to bring any information.

If we fix the graph and the mean-variance ratio, for all the cases $\mathbf{u}_2$ considered, we observe no significant difference in the precision of the variable selection process nor in the extracted correlations. This observation holds for all graph types and mean-to-variance ratios. The correlations between neighbours in the graph did not change the selected variables.

Overall, netSGCCA seemed to outperform the SGCCA in terms of retrieving the variables of interest, in nearly all configurations tested. It appeared that it is through its properties and structures that the graph have an influence on the behaviour of the model. We seek to investigate these results on real oncological data in the next sections.

Table S1: Recovering performances depending on configurations defined by the different cases defined by the vector $\mathbf{u}_2$ and graphs. Corr is the correlation between the estimated components. Precision, Recall and F1 correspond to the evaluation of $\mathbf{u}_2$ against the computed weights. Bold refers to highest values between netSGCCA and SGCCA. Low mean to variance ratio ($c = 0.5$).

			Graph Used				No graph used			
		$\gamma_{\mathcal{G}}$	Corr	Precision	Recall	F1	Corr	Precision	Recall	F1
Case 1	Path	10^{-4}	**0.56 ± 0.05**	0.2 ± 0.11	0.17 ± 0.1	**0.18 ± 0.1**	0.46 ± 0.04	0.22 ± 0.32	0.03 ± 0.03	0.04 ± 0.05
	Star	1	**0.6 ± 0.04**	0.13 ± 0.07	0.3 ± 0.15	**0.18 ± 0.09**	0.48 ± 0.04	0.02 ± 0.09	0.01 ± 0.02	0.01 ± 0.04
	Union	10^{-3}	**0.58 ± 0.05**	0.13 ± 0.07	0.26 ± 0.15	**0.17 ± 0.1**	0.47 ± 0.04	0.09 ± 0.25	0.01 ± 0.03	0.02 ± 0.12
	Complete	10^{-2}	0.39 ± 0.04	0.0 ± 0.0	0.0 ± 0.0	0.0 ± 0.0	**0.4 ± 0.04**	0.0 ± 0.0	0.0 ± 0.0	0.0 ± 0.0
Case 2	Path	10^{-4}	**0.57 ± 0.04**	0.27 ± 0.1	0.22 ± 0.09	**0.24 ± 0.09**	0.47 ± 0.05	0.24 ± 0.32	0.04 ± 0.05	0.06 ± 0.07
	Star	1	**0.6 ± 0.05**	0.2 ± 0.09	0.48 ± 0.24	**0.28 ± 0.13**	0.47 ± 0.05	0.24 ± 0.32	0.03 ± 0.03	0.05 ± 0.06
	Union	10^{-4}	**0.58 ± 0.03**	0.21 ± 0.08	0.43 ± 0.15	**0.29 ± 0.1**	0.47 ± 0.04	0.32 ± 0.39	0.04 ± 0.06	0.07 ± 0.1
	Complete	10^{-1}	0.35 ± 0.04	0.01 ± 0.03	0.01 ± 0.06	**0.01 ± 0.04**	**0.38 ± 0.04**	0.01 ± 0.03	0.0 ± 0.01	0.0 ± 0.02
Case 3	Path	10^{-2}	**0.56 ± 0.04**	0.16 ± 0.07	0.3 ± 0.14	**0.21 ± 0.1**	0.45 ± 0.03	0.1 ± 0.25	0.01 ± 0.02	0.02 ± 0.04
	Star	1	**0.63 ± 0.05**	0.17 ± 0.06	0.37 ± 0.11	**0.24 ± 0.07**	0.49 ± 0.04	0.12 ± 0.15	0.02 ± 0.03	0.03 ± 0.04
	Union	10^{-4}	**0.59 ± 0.03**	0.2 ± 0.05	0.39 ± 0.11	**0.26 ± 0.07**	0.46 ± 0.04	0.16 ± 0.28	0.02 ± 0.03	0.04 ± 0.06
	Complete	10^{-2}	0.39 ± 0.05	0.0 ± 0.0	0.0 ± 0.0	0.0 ± 0.0	0.39 ± 0.05	0.0 ± 0.0	0.0 ± 0.0	0.0 ± 0.0

Table S2: Recovering performances depending on configurations defined by the different cases defined by the vector $\mathbf{u}_2$ and graphs. Corr is the correlation between the estimated components. Precision, Recall and F1 correspond to the evaluation of $\mathbf{u}_2$ against the computed weights. Bold refers to highest values between netSGCCA and SGCCA. High mean to variance ratio ($c = 2$).

			Graph Used				No graph used			
		$\gamma_{\mathcal{G}}$	Corr	Precision	Recall	F1	Corr	Precision	Recall	F1
Case 1	Path	10^{-3}	0.73 ± 0.04	1.0 ± 0.0	1.0 ± 0.0	**1.0 ± 0.0**	**0.76 ± 0.04**	1.0 ± 0.0	0.2 ± 0.05	0.32 ± 0.06
	Star	10^{-4}	0.73 ± 0.05	0.46 ± 0.03	1.0 ± 0.0	**0.63 ± 0.03**	**0.74 ± 0.03**	1.0 ± 0.0	0.21 ± 0.06	0.35 ± 0.07
	Union	10^{-4}	0.72 ± 0.04	0.47 ± 0.05	1.0 ± 0.0	**0.64 ± 0.04**	**0.74 ± 0.04**	1.0 ± 0.0	0.21 ± 0.06	0.35 ± 0.08
	Complete	10^{-4}	0.36 ± 0.09	0.02 ± 0.07	0.06 ± 0.23	0.03 ± 0.11	**0.39 ± 0.09**	0.05 ± 0.22	0.02 ± 0.09	0.03 ± 0.13
Case 2	Path	10^{-3}	0.71 ± 0.04	1.0 ± 0.0	1.0 ± 0.0	**1.0 ± 0.0**	**0.74 ± 0.04**	1.0 ± 0.0	0.24 ± 0.08	0.38 ± 0.11
	Star	10^{-4}	0.7 ± 0.04	0.47 ± 0.03	1.0 ± 0.0	**0.64 ± 0.03**	**0.74 ± 0.04**	1.0 ± 0.0	0.19 ± 0.06	0.32 ± 0.09
	Union	10^{-4}	0.71 ± 0.04	0.47 ± 0.02	1.0 ± 0.0	**0.64 ± 0.02**	**0.74 ± 0.04**	1.0 ± 0.0	0.22 ± 0.05	0.36 ± 0.07
	Complete	1	0.32 ± 0.04	0.02 ± 0.1	0.05 ± 0.22	0.03 ± 0.13	**0.39 ± 0.09**	0.05 ± 0.22	0.02 ± 0.09	0.03 ± 0.13
Case 3	Path	10^{-3}	0.73 ± 0.04	1.0 ± 0.0	1.0 ± 0.0	**1.0 ± 0.0**	**0.74 ± 0.04**	1.0 ± 0.0	0.21 ± 0.06	0.34 ± 0.08
	Star	10^{-4}	0.7 ± 0.05	0.47 ± 0.04	1.0 ± 0.0	**0.64 ± 0.04**	**0.74 ± 0.05**	1.0 ± 0.0	0.2 ± 0.06	0.33 ± 0.08
	Union	10^{-4}	0.71 ± 0.04	0.46 ± 0.04	1.0 ± 0.0	**0.63 ± 0.04**	**0.75 ± 0.03**	1.0 ± 0.0	0.19 ± 0.07	0.31 ± 0.1
	Complete	10^{-4}	0.36 ± 0.09	0.02 ± 0.07	0.07 ± 0.23	**0.03 ± 0.11**	**0.39 ± 0.09**	0.05 ± 0.22	0.01 ± 0.07	0.02 ± 0.1

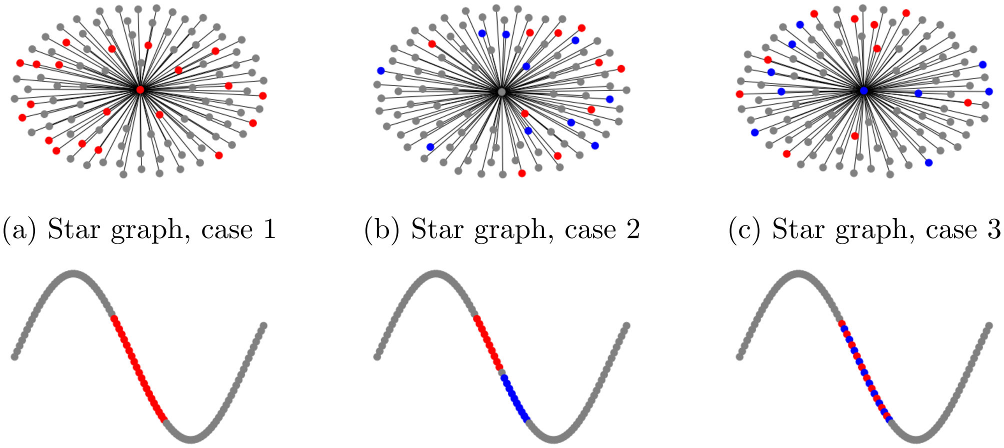

(a) Star graph, case 1 (b) Star graph, case 2 (c) Star graph, case 3

(d) Path graph, case 1 (e) Path graph, case 2 (f) Path graph, case 3

Figure S1: Star and Path graphs with different u_2 values. Grey nodes correspond to 0 values, red for 1, and blue for -1

2 TCGA-LGG: Additional Results

Table S3: Comparison between genes selected by the PC graph and genes selected by the MSIGDB or KEGG.

	# of selected genes	Selected from PC	Size of intersection	Dice	nPOG
MSIGDB	434.51 ± 15	975.37 ± 66	428.33 ± 38	0.61 ± 0.07	0.43 ± 0.05
KEGG	233.78 ± 30	975.37 ± 66	197 ± 65	0.33 ± 0.11	0.20 ± 0.07

Table S4: Comparison between genes selected by the PC graph the 10 permutations of the PC graph

	# of selected genes	Selected from PC	Size of intersection	Dice	nPOG
PERMUTATION1	760.27 ± 27	975.37 ± 66	37.64 ± 5	0.04 ± 0.0	0.00 ± 0.00
PERMUTATION2	848.94 ± 16	975.37 ± 66	43.53 ± 4	0.04 ± 0.0	0.00 ± 0.00
PERMUTATION3	745.36 ± 27	975.37 ± 66	28.74 ± 5	0.03 ± 0.0	0.00 ± 0.00
PERMUTATION4	809.02 ± 30	975.37 ± 66	37.04 ± 4	0.04 ± 0.0	0.00 ± 0.00
PERMUTATION5	826.02 ± 56	975.37 ± 66	37.77 ± 4	0.04 ± 0.0	0.00 ± 0.00
PERMUTATION6	738.57 ± 41	975.37 ± 66	41.10 ± 3	0.04 ± 0.0	0.00 ± 0.00
PERMUTATION7	790.37 ± 41	975.37 ± 66	36.77 ± 4	0.04 ± 0.0	0.00 ± 0.00
PERMUTATION8	812.29 ± 27	975.37 ± 66	40.47 ± 4	0.04 ± 0.0	0.00 ± 0.00
PERMUTATION9	705.63 ± 18	975.37 ± 66	29.34 ± 6	0.03 ± 0.0	0.00 ± 0.00
PERMUTATION10	789.86 ± 46	975.37 ± 66	39.76 ± 4	0.04 ± 0.0	0.00 ± 0.00

Table S5: The effect of pruning edges that connect genes selected when using the full PC graph. Results obtained on 100 samples.

Edges removed	# of selected genes	Selected from PC	Size of intersection	Dice
Inner	936 ± 74	975.37 ± 66	925 ± 111	0.97 ± 0.09
Outer	426 ± 59	975.37 ± 66	426 ± 59	0.61 ± 0.03
Inner and outer	169 ± 17	975.37 ± 66	165 ± 8	0.29 ± 0.02

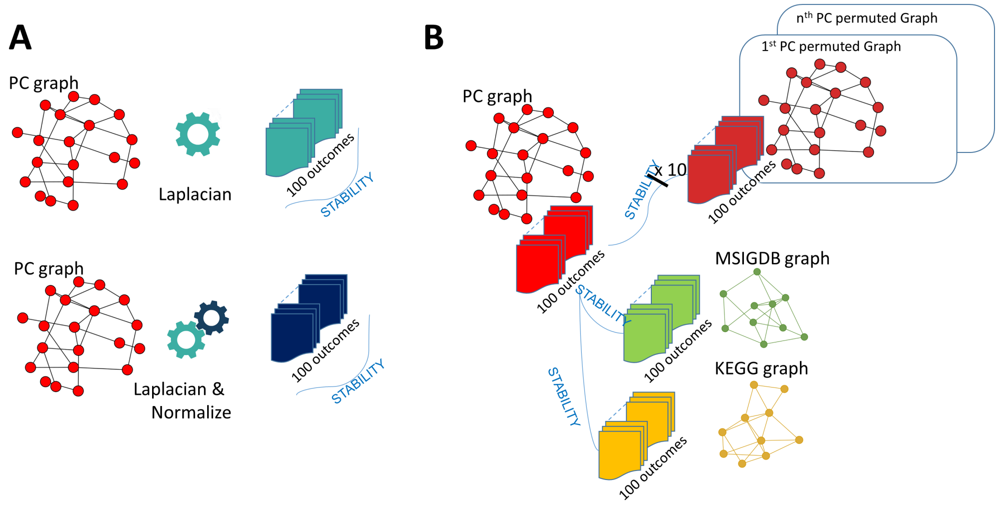

Figure S2: Diagram showing the basis of the stability analyses performed in paragraphs 4.1 and 4.2. A. panel: from the n=100 outcomes of the runs performed on the 100 bootstrap samples we computed DICE and nPOG metrics on all different pairs of outcomes out of the one hundred ones. From these (n.(n-1)/2) DICE and nPOG values are derived mean and standard deviation. The stability study was done in the configurations when only the raw graph Laplacian or the normalised graph Laplacian was used. B. panel: the (PathwayCommon) PC-graph is considered as reference. The stability is computed between PC and each of the MSIGDB, KEGG, Permuted1,... and Permuted10 graphs. In each cases, n paired outcomes are used to derive a mean and standard deviation of DICE and nPOG.

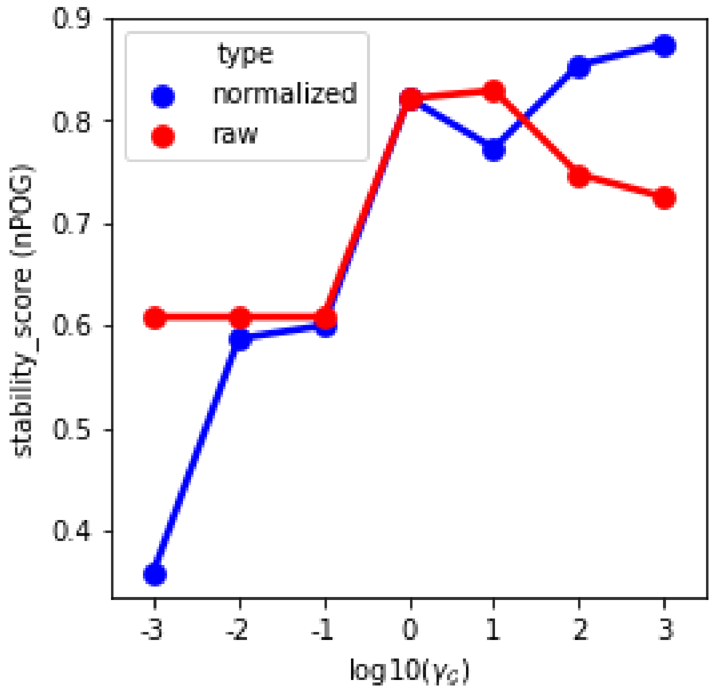

Figure S3: Evolution of the nPOG metric as $\gamma_{\mathcal{G}}$ varies, using the raw and normalised graph Laplacian.

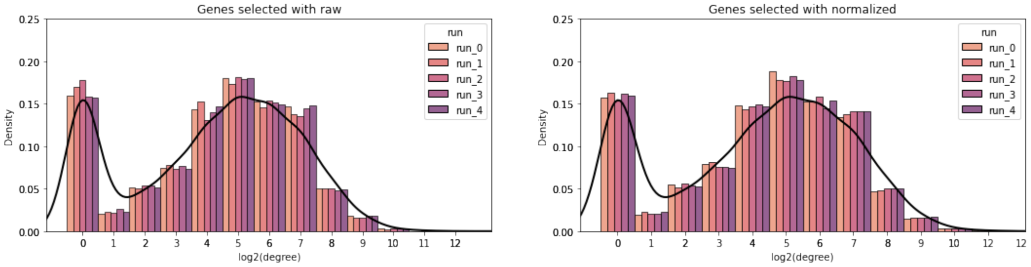

(a)

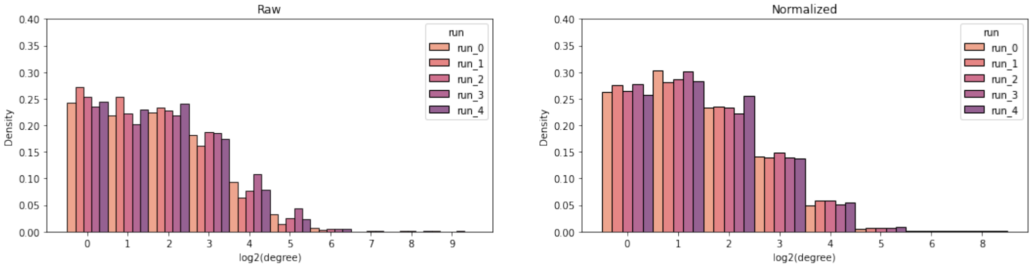

(b)

Figure S4: Degree distribution of selected genes by fold for Raw and Normalised graph Laplacians. (a) For each selected gene, we counted the number of its neighbours in the PC graph. The black line represents the density of the degree distribution of all genes in the PC graph. (b) For each selected gene, we counted the number of its neighbours among selected genes.

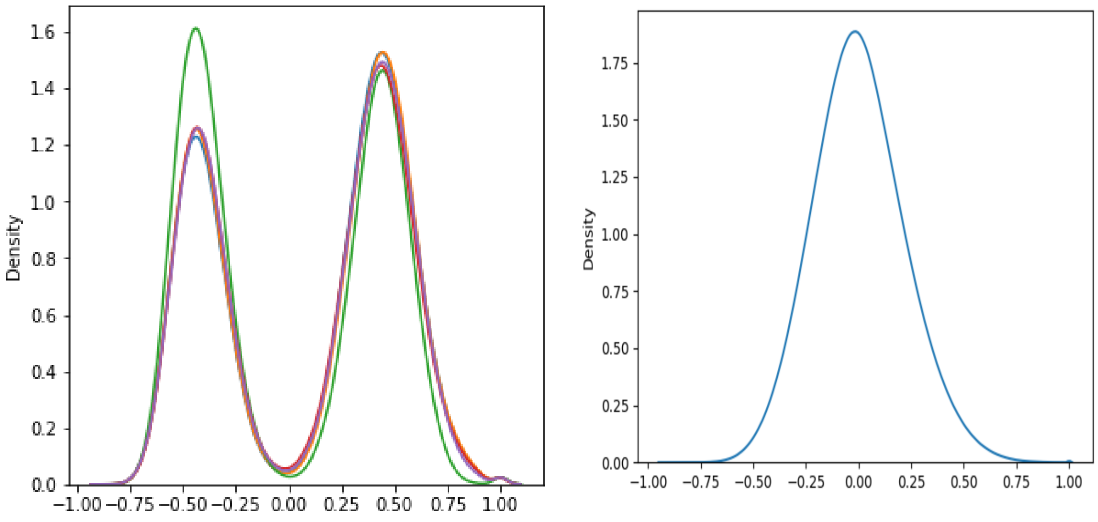

(a) (b)

Figure S5: (a) Correlation distribution of selected genes from 5 random runs. (b) Correlation distribution of all genes in the dataset

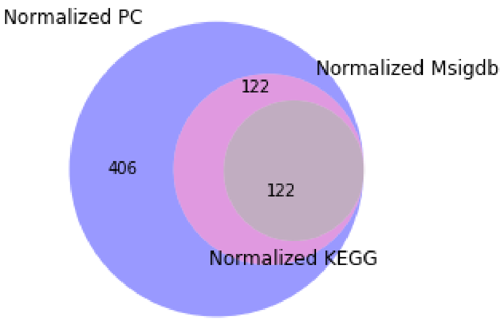

Figure S6: Venn diagram showing the overlap between genes selected by the PC graph and the MSIGDB and the KEGG Graphs. Only genes selected in over 80% of runs were used.

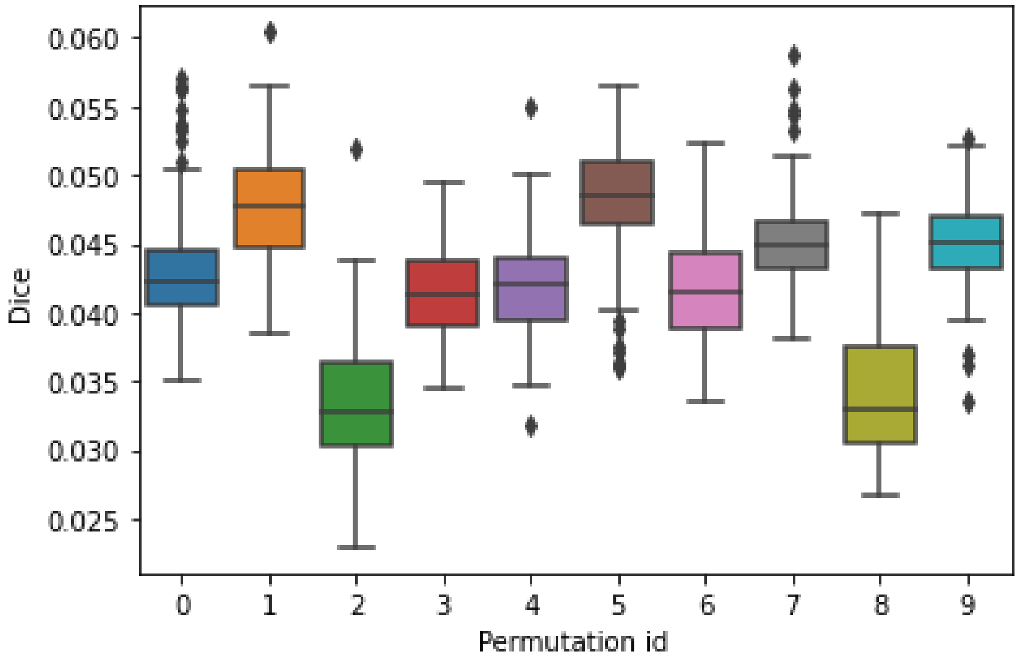

Figure S7: Box plot for the Dice metric between the genes selected by the PC graph and the permuted PC graph for each run. Results were separated for each permutation

3 Additional datasets

To verify the robustness of the presented results, we analysed three other oncological datasets using the same experimental design as on the TCGA-LGG. These datasets comprise the TCGA-KIRP dataset of 167 patients diagnosed with kidney renal papillary cell carcinoma, the pancreatic adenocarcinoma dataset TCGA-PAAD with 124 patients, and the TCGA-OV dataset of 219 patients diagnosed with ovarian serous cystadenocarcinoma. These datasets differ regarding tumour location, sample sizes, survival profiles, and event rates, as shown in table S6.

Table S6: Description of the different datasets used

	tumour location	sample size	event rate
TGCA-LGG	brain	419	0.18
TCGA-KIRP	kidney	167	0.12
TCGA-PAAD	pancreas	124	0.42
TCGA-OV	ovaries	219	0.50

For each dataset, we compared the raw and normalised graph Laplacian using the Pathway Commons graph and in terms of variable selection and stability, as done earlier. Additionally, we also analysed the effect of the removal of graph edges.

3.1 Comparison between normalised and raw graph Laplacian

Looking at the stability of the models using the Dice metric, as exhibited in Figure S9, the stability increases as $\gamma_{\mathcal{G}}$ grows when the normalised graph Laplacian is used. In contrast, the stability using the raw graph Laplacian will often decrease sharply for high values of $\gamma_{\mathcal{G}}$. Out of the four datasets used in this work, only on TCGA-PAAD did the raw graph Laplacian outperform the normalised graph Laplacian when $\gamma_{\mathcal{G}}$ is greater than 1. However, the stability ranges differ between the different datasets, which shows that obtained results are data-dependent.

Figure S10 the distribution of the selection rate of the genes selected at least once in the 100 runs, with $\gamma_{\mathcal{G}} = 10^3$. This distribution is data-dependent. However, most genes are selected a few times. On the TCGA-

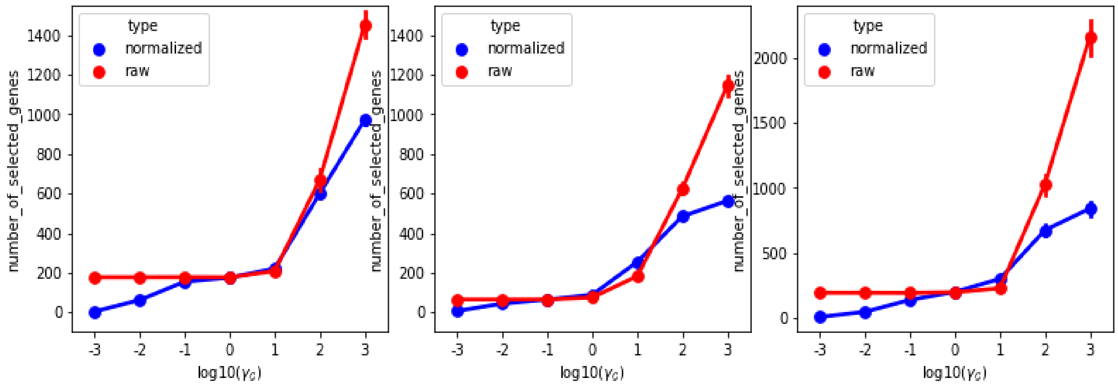

(a) (b) (c)

Figure S8: Evolution of the number of selected genes as $\gamma_\mathcal{G}$ varies, using the raw and normalised graph Laplacian. (a) TCGA-KIRP, (b) TCGA-PAAD, (c) TGCA-OV.

PAAD and TCGA-OV, the distribution is reflected in the low stability of the models, as shown previously. On the TCGA-KIRP, we obtained higher dice stability scores when the normalised graph Laplacian was used, which is explained by a better distribution of the selection rate.

Figure S8 shows that the number of selected variables increases as $\gamma_\mathcal{G}$ grows on the different studied datasets. This is in line with our findings on the TCGA-LCC dataset. Again, the increase is smoother when the normalised graph Laplacian is used.

As was previously done for TCGA-LGG, Figure S11 shows the degree distribution of selected genes, on the whole graph and the sub-graph. Both Figures exhibit similar patterns between all the studied datasets, demonstrating that our observations from the TCGA-LGG hold across datasets. Namely, the graph penalty does not favour highly connected notes, nor does it select sub-regions from the graph.

3.2 Comparisons between different graphs

Again, we permuted the notes in the graph to study the effects of the graph semantics while keeping the same graph structure. The low Dice scores shown in Figure S13 indicate little consistency between the results obtained using the original PC and permuted graphs. This is in line with results found on

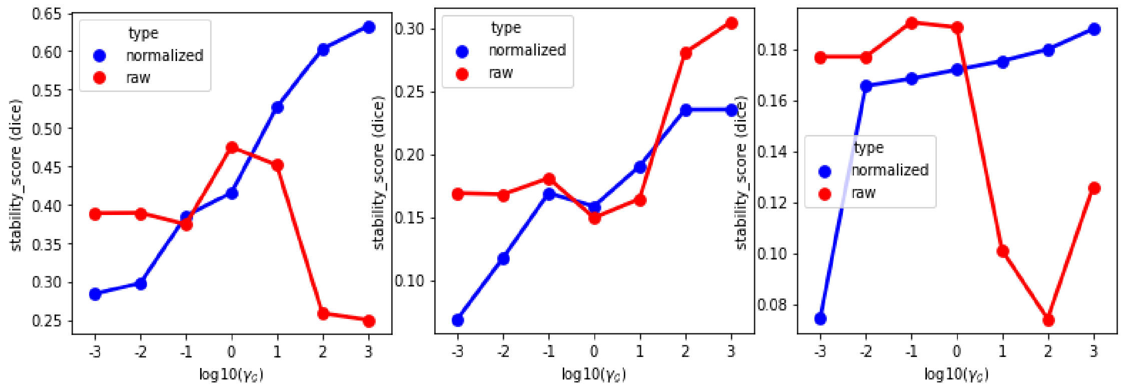

(a) (b) (c)

Figure S9: Evolution of the Dice metric as $\gamma_{\mathcal{G}}$ varies, using the raw and normalised graph Laplacian. (a) TCGA-KIRP, (b) TCGA-PAAD, (c) TGCA-OV.

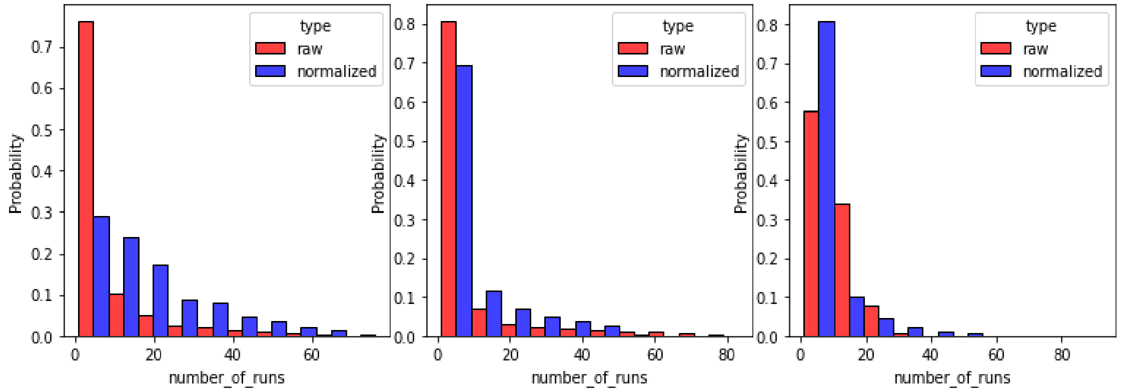

(a) (b) (c)

Figure S10: Distribution of the selection rate of the genes selected at least once in the 100 runs, with $\gamma_{\mathcal{G}} = 10^3$. (a) TCGA-KIRP, (b) TCGA-PAAD, (c) TGCA-OV.

TCGA-LGG and demonstrates that the semantics of the chosen graph have a substantial impact on feature selection.

Having a selected set of genes, we define inner edges as the direct links between the selected genes, and outer edges as links between a selected gene

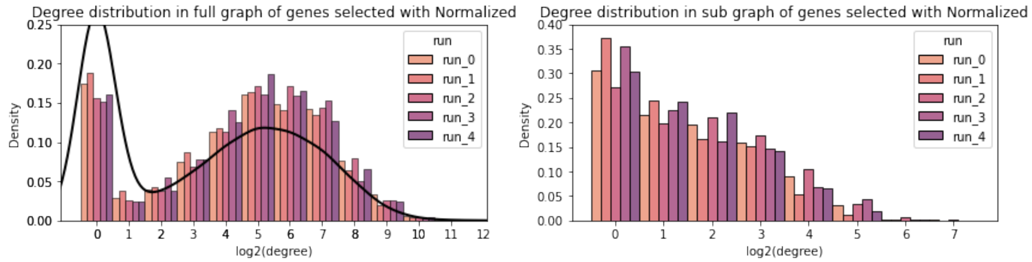

(a)

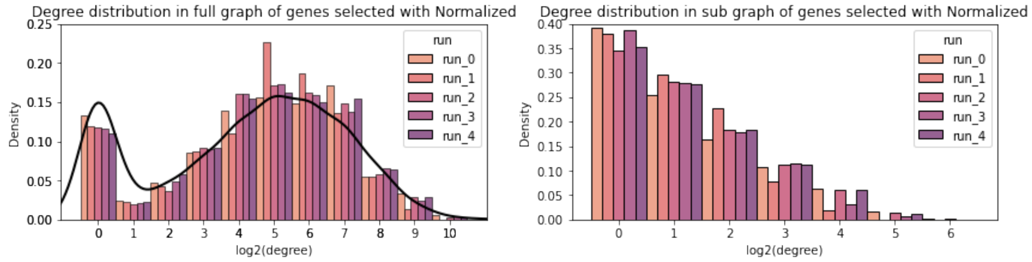

(b)

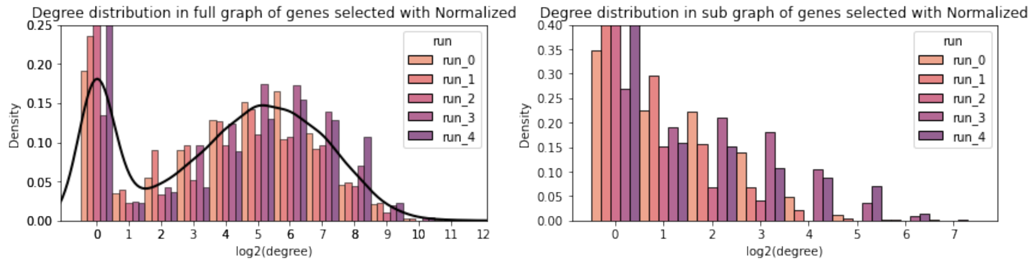

(c)

Figure S11: Degree distribution of selected genes in five random runs for normalised graph Laplacian. On the left, for each selected gene, we counted the number of its neighbours in the PC graph. The black line represents the density of the degree distribution of all genes in the PC graph. On the right, for each selected gene, we counted the number of its neighbours among selected genes. (a) TCGA-KIRP, (b) TCGA-PAAD, (c) TGCA-OV.

and a not-selected gene. For each of the 100 runs, and on each dataset, we investigated the impact of removing each set of edges on the variable selection. As done on the TCGA-LGG dataset, we compared the results obtained on each run when the original Pathway Commons graph and its pruned version

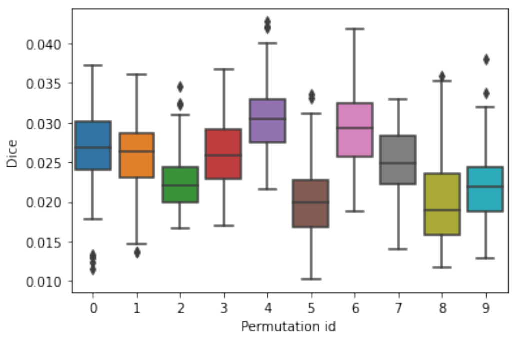

(a)

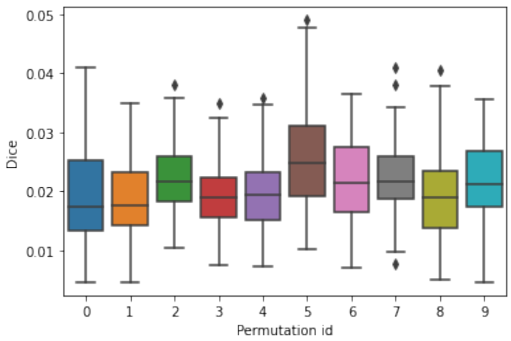

(b)

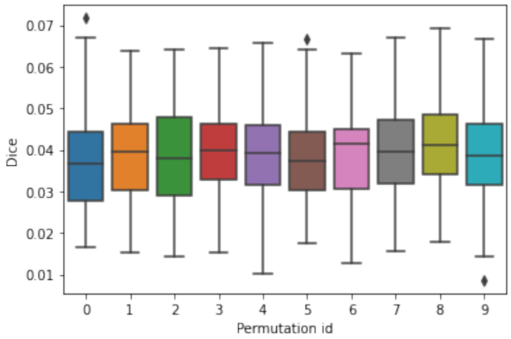

(c)

Figure S12: Box plot for the Dice metric between the genes selected by the PC graph and the permuted PC graph for each run. Results were separated for each permutation. (a) TCGA-KIRP, (b) TCGA-PAAD, (c) TGCA-OV.

were used. Results are shown in Table S7. As seen on TCGA-LGG, removing direct edges between selected variables did not significantly impact variable selection. However, making selected variables isolated considerably reduced the number of selected variables. The existence of paths between variables is important for the variable selection process.

Finally, we randomly removed edges in the graph and analysed the number of selected variables. On all datasets, including TCGA-LGG, the number of selected variables follows the decrease in the number of the graph edges, thus graph density.

Table S7: The effect of pruning edges that connect genes selected when using the full PC graph. Results obtained on 100 samples, on TCGA-KIRP, TCGA-PAAD, TCGA-OV.

Dataset	Edges removed	# of selected genes	Selected from PC	Size of intersection	Dice
TCGA-KIRP	Inner	962 ± 92	972 ± 95	928 ± 147	0.96 ± 0.13
	Outer	411 ± 63	972 ± 95	394 ± 49	0.57 ± 0.05
	Inner and outer	180 ± 34	972 ± 95	171 ± 10	0.30 ± 0.03
TCGA-PAAD	Inner	559 ± 74	563 ± 74	551 ± 86	0.98 ± 0.08
	Outer	196 ± 121	563 ± 74	131 ± 44	0.36 ± 0.12
	Inner and outer	71 ± 23	563 ± 74	66 ± 7	0.21 ± 0.04
TCGA-OV	Inner	815 ± 273	841 ± 282	742 ± 325	0.89 ± 0.24
	Outer	543 ± 173	841 ± 282	401 ± 177	0.58 ± 0.19
	Inner and outer	259 ± 142	841 ± 282	182 ± 43	0.35 ± 0.12

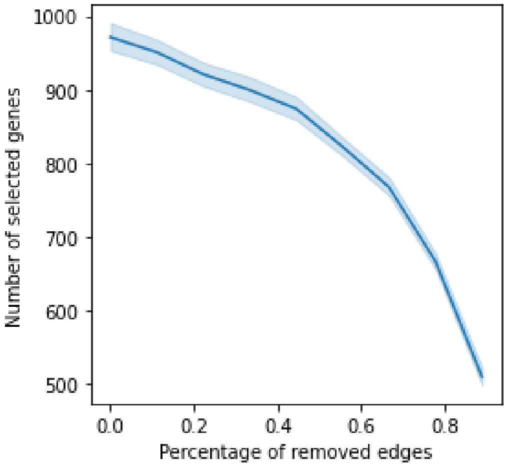

(a)

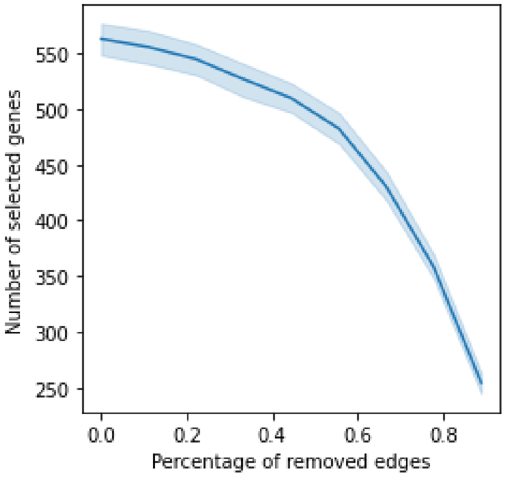

(b)

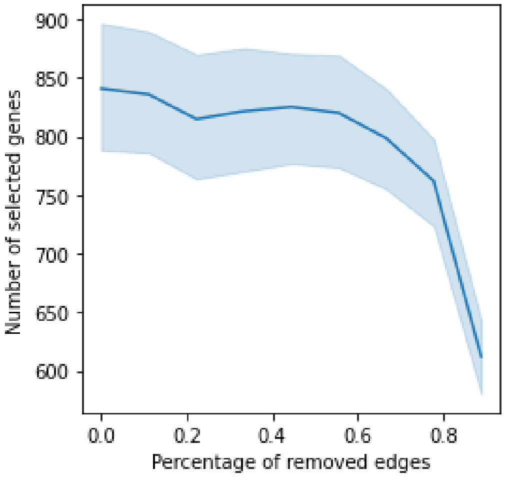

(c)

Figure S13: The evolution of the number of selected genes when the number of edges decreases. (a) TCGA-KIRP, (b) TCGA-PAAD, (c) TGCA-OV.

References

[1] Lei Du, Kefei Liu, Xiaohui Yao, Shannon L. Risacher, Junwei Han, Andrew J. Saykin, Lei Guo, and Li Shen. Detecting genetic associations with brain imaging phenotypes in Alzheimer's disease via a novel structured SCCA approach. *Medical Image Analysis*, 61:101656, April 2020. ISSN 1361-8415. doi: 10.1016/j.media.2020.101656. URL `https://www.sciencedirect.com/science/article/pii/S1361841520300232`.

[2] Nicholas J. Higham. Computing the nearest correlation matrix—a problem from finance. *IMA Journal of Numerical Analysis*, 22(3):329–343, 2002. doi: 10.1093/imanum/22.3.329.
